# Predisposing and precipitating risk factors for delirium in gastroenterology and hepatology: Subgroup analysis of 718 patients from a hospital-wide prospective cohort study

**DOI:** 10.3389/fmed.2022.1004407

**Published:** 2022-11-30

**Authors:** Florian F. Hildenbrand, Fritz R. Murray, Roland von Känel, Ansgar R. Deibel, Philipp Schreiner, Jutta Ernst, Carl M. Zipser, Soenke Böettger

**Affiliations:** ^1^Department of Gastroenterology and Hepatology, University Hospital, University of Zurich, Zurich, Switzerland; ^2^Department of Gastroenterology and Hepatology, Stadtspital Zurich, Zurich, Switzerland; ^3^Department of Consultation-Liaison Psychiatry and Psychosomatic Medicine, Zurich, Switzerland; ^4^Center of Clinical Nursing Science, University of Zurich, University Hospital of Zurich, Zurich, Switzerland; ^5^Department of Neurology and Neurophysiology, Balgrist University Hospital, University of Zurich, Zurich, Switzerland

**Keywords:** delirium, hepatic encephalopathy (HE), gastroenterology, hepatology, predisposing and precipitating risk factors

## Abstract

**Background and Aims:**

Delirium is the most common acute neuropsychiatric syndrome in hospitalized patients. Higher age and cognitive impairment are known predisposing risk factors in general hospital populations. However, the interrelation with precipitating gastrointestinal (GI) and hepato-pancreato-biliary (HPB) diseases remains to be determined.

**Patients and methods:**

Prospective 1-year hospital-wide cohort study in 29’278 adults, subgroup analysis in 718 patients hospitalized with GI/HPB disease. Delirium based on routine admission screening and a DSM-5 based construct. Regression analyses used to evaluate clinical characteristics of delirious patients.

**Results:**

Delirium was detected in 24.8% (178/718). Age in delirious patients (median 62 years [IQR 21]) was not different to non-delirious (median 60 years [IQR 22]), *p* = 0.45). Dementia was the strongest predisposing factor for delirium (OR 66.16 [6.31–693.83], *p* < 0.001). Functional impairment, and at most, immobility increased odds for delirium (OR 7.78 [3.84–15.77], *p* < 0.001). Patients with delirium had higher in-hospital mortality rates (18%; OR 39.23 [11.85–129.93], *p* < 0.001). From GI and HPB conditions, cirrhosis predisposed to delirium (OR 2.11 [1.11–4.03], *p* = 0.023), while acute renal failure (OR 4.45 [1.61–12.26], *p* = 0.004) and liver disease (OR 2.22 [1.12–4.42], *p* = 0.023) were precipitators. Total costs were higher in patients with delirium (USD 30003 vs. 10977; *p* < 0.001).

**Conclusion:**

Delirium in GI- and HPB-disease was not associated with higher age *per se*, but with cognitive and functional impairment. Delirium needs to be considered in younger adults with acute renal failure and/or liver disease. Clinicians should be aware about individual risk profiles, apply preventive and supportive strategies early, which may improve outcomes and lower costs.

## Introduction

Delirium is the most common acute neuropsychiatric syndrome in general hospital populations with a substantial impact on individual health and public economy (1). The current Diagnostic and Statistical Manual of Mental Disorders (DSM-5) characterizes delirium by disturbances in consciousness, cognition, and a range of non-cognitive domains, in addition to an abrupt onset and fluctuating course (2). In the general hospital setting, the overall delirium prevalence rates in non-surgical patients ranges from 18 to 35% (1, 3). Most often, the aetiology of delirium is multifactorial, with metabolic, drug, infectious and organ-specific diseases (and/or interactions of those) leading to an alteration in neurotransmitter synthesis and function, along with dysregulation of neuronal activity, subsequently leading to brain dysfunction (1). The sequelae of delirium are severe and include short-term (e.g., increased morbidity and mortality, or prolonged hospitalization) and long-term consequences, such as cognitive decline, deterioration in functionality and institutionalization (4). Presence and severity of delirium can be measured with several high-quality instruments, for instance, with Delirium Observation Screening Scale (DOS) (5). Risk factors are divided into predisposing and precipitating ones, as well as potentially modifiable and non-modifiable factors (6–8). Across most services, predisposing factors include higher age, cognitive and functional impairment (such as malnutrition and frailty) (9, 10). Precipitating risk factors can be differentiated to primary cerebral conditions, e.g., stroke or intracranial hemorrhage (11–13), and systemic (secondary) conditions, e.g., postoperative delirium in orthopedic surgery or sepsis (14, 15). Predisposing and precipitating factors interact: in patients with low predisposition, the precipitating factors or noxious insults have to be more severe than in patients with high predisposition, in which less severe noxious insults cause delirium (6). In patients with gastrointestinal (GI) and hepato-pancreato-biliary (HPB) diseases, several studies investigated hepatic encephalopathy specifically (16), however, the risk profile of these patients remained understudied. Therefore, the aim of this study was to assess predisposing and precipitating risk factors in a subgroup of GI- and HPB-patients from an observational cohort study conducted in an acute hospital setting of a tertiary university hospital in Switzerland.

## Materials and methods

### Patients and procedures

**FIGURE 1:**
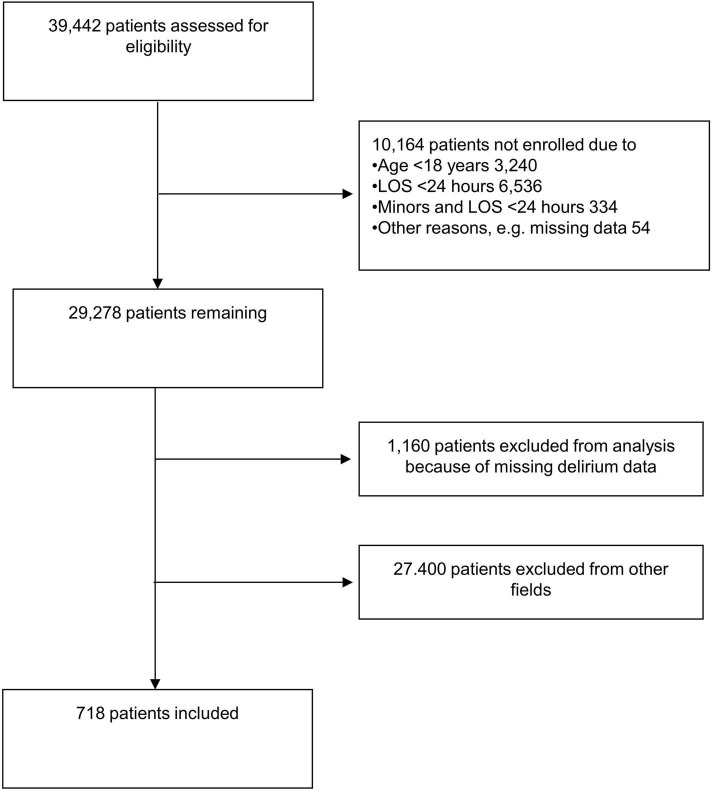
Flow diagram of patient selection. LOS, length of stay.

All data described in this prospective cohort study was obtained in DELIR-PATH (Detect Evaluate Control Inpatient Risk factors, Prevent And Treat Hospital Acquired Deliriums), a quality improvement project aiming to investigate the detection and management of delirium in hospitalized patients at the University Hospital Zurich, a large 900-bed tertiary care center (17). The delirium screening data was collected over a time period of 12 months and followed the STROBE standard (17). Over one year (2014), 39,442 patients were admitted and consecutively enrolled in the DELIR-PATH project. Exclusion criteria were age < 18 years, length of stay (LOS) < 24 h, as well as missing data, leaving 29,278 eligible patients. Out of these, 718 patients were managed by the gastroenterological service and were included in this study (Figure 1). Relevant data on medical diagnoses and functional impairment for this sub-study was automatically retrieved from the electronic medical chart (KLINIKINFORMATIONSSYSTEM, KISIM, CISTEC AG, ZURICH) and a 56-item nursing instrument (EPA-AC, epaCC GmbH, Wiesbaden). All study procedures were in accordance with the World Health Organization’s Declaration of Helsinki (18). The study protocol was approved by the ethics committee of the Canton of Zurich and in compliance with Swiss federal law, a waiver of informed consent was granted by the ethics committee (KEK-ZH-Nr. PB-2016-01264).

### Determination of delirium

For the purpose of this study, delirium was determined with DOS, DSM-5 and the ePA-AC, which were obtained by specially trained nursing staff (2, 19, 20). A patient was considered delirious if any of these tools indicated the presence of delirium at least once during the hospitalization (*ila nanila*). This delirium construct was compared with the reference standard delirium diagnoses as determined by a consultation-liaison psychiatrist in a former study and has shown comparable characteristics among the screening approaches (21). It resulted in a delirium prevalence comparable to the recent literature. This construct accurately detects delirium in 97% of cases (22).

### Data processing

For this analysis, diagnostic clusters according to the 10th revision of the International Classification of Diseases (ICD-10) were constructed. To achieve the most correct description of clusters representing the predisposing and precipitating factors, redundant clusters from different chapters were collapsed, e.g., dementias included the psychiatric F00-03 cluster and the neurological cluster- G30-32 “other degenerative disorders of the central nervous system” (DCD) (refer Supplementary Table 1). Cirrhosis was considered a predisposing factor for the development of delirium (4, 6, 8). Finally, nine clusters were included in the analysis: five medical (dementia, substance use, cirrhosis, liver disease, and acute renal failure) and four functional (impairment of activity, mobility, hearing, and vision). We further identified specific clusters for the development of delirium and for increased risk of death with logistic regression. Then, functional impairment, as an approximation of the geriatric concept of frailty, characterized by diminished strength, endurance, and physiological functions, was defined with ePA-AC item “activity” and was dichotomized in regular mobility versus either reduced, assisted or no mobility (23). Further outcome parameters were the number of nursing hours per case, which were evaluated once per shift, and the overall costs per case (CHF, converted to USD using the 2014 OECD purchasing power parity (rate of currency conversion: 1.28) (24).

### Statistical methods

Data was analysed with the Statistical Package for the Social Sciences (SPSS). Descriptive data is presented as means and standard deviations or median and interquartile ranges for ordinal and continuous variables, and as size and percentages for nominal variables. The normality of the data distribution was tested with the Shapiro-Wilk test. Inter-group differences for continuous variables were computed using Student’s *t*- and Mann–Whitney U-test and for categorical variables with Pearson’s-χ^2^ or Fisher’s exact test, where appropriate. The data set was dichotomized according to the presence or absence of delirium. Further dichotomization was made on age (≥ 65 vs. < 65 years). Costs per case and nursing hours were compared using quantile regression.

Patient characteristics and trajectories were determined with simple logistic regressions and their corresponding odds ratios (ORs) and 95% confidence intervals (CIs). For the evaluation of the impact of individual diagnostic clusters, a similar approach with multiple logistic regressions were calculated, with the dependent variable set on the presence or absence of delirium and the respective diagnoses clusters treated as covariates. The model was optimized with Cox-Snell’s and Nagelkerke’s r^2^. The level of significance was set at *p* < 0.05.

## Results

### Baseline characteristics

The overall rate of delirium in this cohort was 24.8%. As shown in Table 1, there were no relevant differences between delirium and non-delirium patients in terms of being ≥ 65 years of age or sex distribution. In the delirium group (*n* = 178), 39.9% had liver disease (LD), 18% upper gastrointestinal tract disease (GI), and 10% malignancy.

**TABLE 1:** Patient baseline characteristics.

	Patients with delirium (*N* = 178)	Patients without delirium (*N* = 540)	P. OR. CI
Age in years*	62 (21)	60 (22)	0.45
> 65 years (%)^#^	76 (42.7)	214 (39.6)	0.48, 1.14. 0.81–1.6
Sex(%)^#^ Male Female	66.3 33.7	59.6 40.4	0.13, 1.33. 0.93–1.9 0.13, 0.75. 0.53–1.07
Main diagnosis (*N*.%)^#^ Pancreato-biliary disease Liver disease Upper gastrointestinal disease Lower gastrointestinal disease Inflammatory bowel disease Functional/Irritable bowel disease Infectious disease Malnutrition Malignancy Other	10 (5.6) 72 (39.9) 32 (18) 9 (5.1) 2 (1.1) 5 (2.8) 13 (7.3) 3 (1.7) 18 (10.1) 14 (7.9)	103 (19) 86 (15.9) 101 (18.7) 30 (5.6) 34 (6.3) 18 (3.3) 28 (5.1) 6 (1.1) 108 (20) 26 (4.8)	<0.001, 0.25. 0.13–0.5 <0.001, 3.59. 2.46–5.23 0.83, 0.95. 0.61–1.48 0.85, 0.91. 0.42–1.95 0.009, 0.17. 0.4–0.71 0.81, 0.84. 0.31–2.29 0.35, 1.44. 0.73–2.85 0.6, 1.53. 0.38–6.27 0.003, 0.45. 0.27–0.77 0.13, 1.69. 0.86–3.31
Dementia/Degenerative cerebral disorders (*N*.%)^#^	13 (7.3)	1 (0.2)	<0.001, 42.47. 5.51–327.1
Admitted from (%)^#^ Home Assisted living Outside hospital	65.2 9 27	85.4 1.5 12.6	<0.001, 0.32. 0.22–0.47 <0.001, 6.57. 2.76–15.63 <0.001, 2.56. 1.69–3.89
Admission mode (%)^#^ Emergent admission Elective admission	73 18	58.3 39.8	<0.001, 1.94. 1.33–2.81 <0.001, 0.37. 0.22–0.51
Length of stay in days (Mean. CI)	20.1 (16–24.2)	7.2 (6.5–7.9)	<0.001
Intensive care management (%)^#^	21.3	1.9	<0.001, 14.39. 6.99–29.59
Discharged to (%)^#^ Home Assisted living Outside hospital Rehabilitation	53.9 6.2 11.2 9	93.5 1.9 3 1.1	<0.001, 0.08. 0.05–0.13 0.008, 3.49. 1.46–8.37 <0.001, 4.15. 2.1–8.19 <0.001, 8.79. 3.38–22.83
Deceased (*N*.%)^#^	32(18)	3 (0.6)	<0.001, 39.23. 11.85–129.93
Costs per case USD^&^	30003 (2906–711677)	10977 (3299–182190)	<0.001

*P* < 0.05 was assumed statistically significant. ^#^The percentages refer to the proportion of the respective group (delirium vs. non-delirium). *Values displayed as median and interquartile range. ^&^Values showed as median and range. OR, Odds ratio; CI, confidence Interval; USD, US-Dollar.

Main admission diagnoses were LD (22%), upper GI-disease (19%), malignancy (18%) and pancreato-biliary (PB) disease (16%). 29% of individuals in the entire cohort suffered from cirrhosis. A detailed classification of the underlying ICD-10 diagnoses and the etiologies of the various diseases considered can be found in the Supplementary material (refer Supplementary Table 2, Supplementary Figures 1 and 2).

**FIGURE 2:**
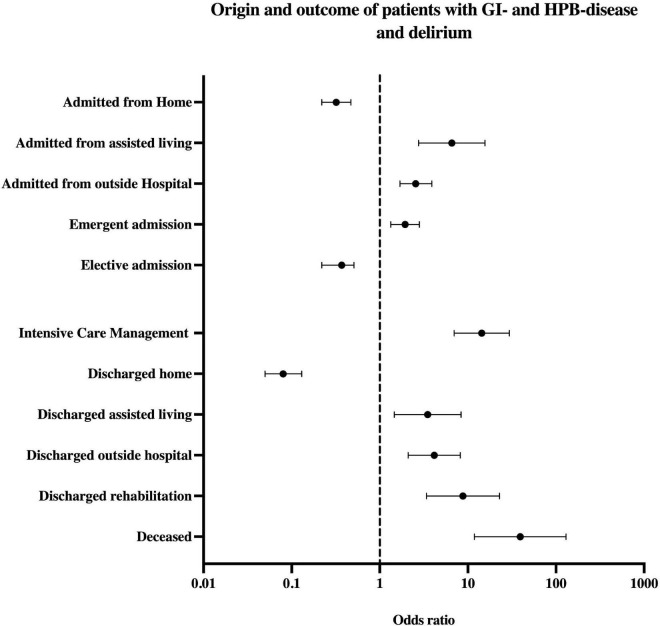
Odds ratio by origin and outcome parameters of patients with delirium and GI-/HPB-disease. The upper part shows the origin before hospital admission and admission mode, below section transfer type and outcome. HPB, hepato-pancreato-biliary.

Patients developing delirium were more commonly admitted from assisted living (OR 6.57, *p* < 0.001, CI 2.76–15.63), other hospitals (OR 2.76, *p* < 0.001, CI 1.69–3.89) and as emergent admission (OR 1.94, *p* < 0.001, CI 1.33–2.81) (refer Table 1 and Figure 2). Patients with delirium stayed on average three times longer in hospital (20.1 vs. 7.7 days) and more frequently required intensive care (ICU) (OR 14.39, *p* < 0.001, CI 6.99–29.59) (see Figure 2 and Table 1). Patients with delirium were more commonly discharged to assisted living (OR 3.49, *p* = 0.005, CI 1.46–8.37), rehabilitation (OR 8.79, *p* < 0.001, CI 3.13–21.45), or to secondary care hospitals (OR 4.15, *p* < 0.001, CI 2.1–8.19) and less commonly returned home (OR 0.08, *p* < 0.001, CI 0.05–0.13).

The median total costs per case were 173% higher in the delirium group, with a significantly higher nursing workload (166 vs. 31 nursing hours), leading to higher median total costs per case (23,440CHF vs. 8,576 CHF or 30,003 USD vs. 10,977 USD).

### Predisposing and precipitating factors for delirium

**FIGURE 3:**
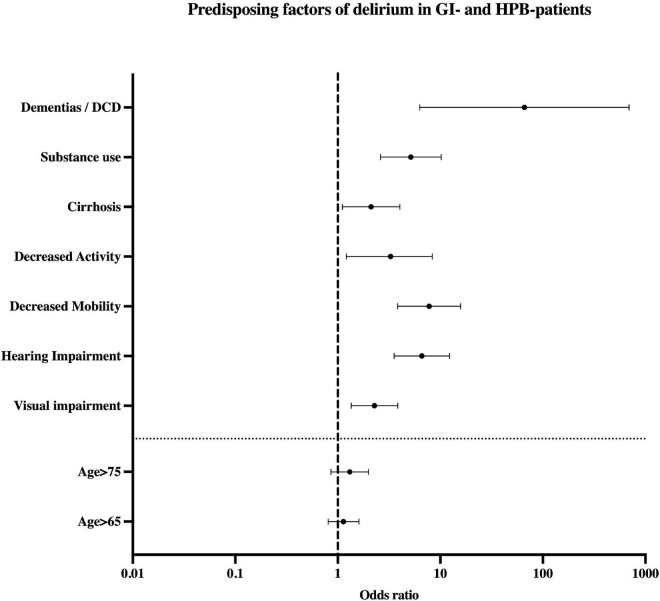
Predisposing factors of delirium in patients with GI-/HPB-disease. Seven clusters were included in the analysis: three medical (Dementias/DCD, Substance use, Cirrhosis) and four functional (decreased activity, mobility, hearing impairment and visual impairment). Values displayed as Odds ratio. DCD, degenerative cerebral diseases; HPB, hepato-pancreato-biliary.

**FIGURE 4:**
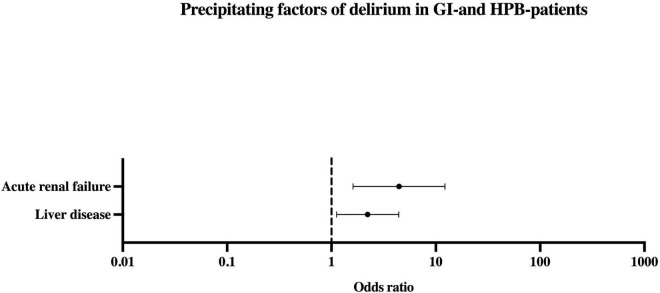
Precipitating factors of delirium in patients with GI-/HPB-disease. 2 Medical clusters have been identified as precipitating factors (Acute renal failure and Liver disease). Values displayed as Odds ratio. HPB, hepato-pancreato-biliary.

Predisposing factors for delirium were decreased activity (OR 3.27, *p* = 0.02, CI 1.21–8.84) or mobility (OR 7.78, *p* < 0.001, CI 3.84–15.77), and impaired hearing (OR 6.6, *p* < 0.001, CI 3.54–12.31) or vision (OR 2.28, *p* = 0.002, CI 1.35–3.85). Same was true for pre-existent dementia and other DCD (OR 66.2, *p* < 0.001, CI 6.31–693.83), substance use (OR 5.16, *p* < 0.001, CI 2.61–10.2) and cirrhosis (OR 2.11, *p* = 0.023, CI 1.11–4.03). Age, especially age ≥ 65 years, did not predict delirium (refer Table 2 and Figure 3). As shown in Table 2 and Figure 4, precipitating factors associated with delirium were LD (OR 2.22, *p* = 0.023, CI 1.12–4.42) and acute renal failure (ARF) (OR 4.45, *p* = 0.004, CI 1.61–12.26). In the remaining subgroups according to ICD-10, there was no significant risk for developing delirium (refer Supplementary Figure 2). When both precipitating factors occurred in combination, the odds ratio of delirium increased to 26.57 (*p* < 0.001, CI 6.05–116.76). Presence of ARF in addition to the predisposing factor cirrhosis yielded in an OR for delirium of 64.41 (*p* < 0.001, CI 8.56–484.88).

**TABLE 2:** Multiple regression model for predisposing and precipitating factors of delirium in GI-/HPB-patients.

	B (SE)	Exp (B)	95% CI lower – upper	*P*
**Predisposing factors**				
Activity impairment	*1.19 (0.51)*	*3.27*	*1.21*–*8.34*	***0.020***
Mobility impairment	*2.05 (0.36)*	*7.78*	*3.84*–*15.77*	< ***0*.*001***
Hearing impairment	*1.89 (0.32)*	*6.6*	*3.54*–*12.31*	< ***0*.*001***
Visual impairment	*0.82 (0.27)*	*2.28*	*1.35*–*3.85*	***0.002***
Dementia	*4.19 (1.2)*	*66.16*	*6.31*–*693.83*	< ***0*.*001***
Substance use	*1.64 (0.35)*	*5.16*	*2.61*–*10.2*	< ***0*.*001***
Cirrhosis	*0.75 (0.33)*	*2.11*	*1.11*–*4.03*	***0.023***
**Precipitating factors**				
Liver disease	*0.8 (0.35)*	*2.22*	*1.12*–*4.42*	***0.023***
Acute renal failure	*1.49 (0.52)*	*4.45*	*1.61*–*12.26*	***0.004***
Constant	–5.07 (0.47)	0.006		< ***0*.*001***

The population was dichotomized into delirious and non-delirious. Odds ratios (OR) > 1 indicate a strong association with the patient group (when compared with the variable reference level) whereas ORs < 1 represent no association with the patient group (when compared with the variable reference level). Cox–Snell and Nagelkerke r2 = 0.413 and r2 = 0.613. Displayed are estimated coefficients (B, SE), 95% confidence intervals, and P-values. Bold values indicated significant results. SE, standard error; CI, confidence interval. B: estimated coefficient, Exp (B): exponential value of B corresponds to odds ratio, P: P-value.

### Risk of death

32/178 patients (18%) with versus 3/540 (0.6%) without delirium died during hospitalization, reflected by substantially increased odds ratio for death (OR 39.23, *p* < 0.001, CI 11.85–129.93). The occurrence of LD and ARF in delirium resulted in an OR for in-hospital mortality of 10.18 (*p* < 0.001, CI 3.33–31.16). This risk of death in patients with ARF, cirrhosis, and delirium was 18.10 (*p* < 0.001, CI 6.74– 48.65).

## Discussion

### Summary of main findings

Patients with GI- and HPB- diseases and delirium were hospitalized longer, had higher mortality rates, and need for acute rehabilitation or assisted living at discharge. Total costs and nursing hours were higher in these patients. The strongest predisposing factors were dementia and functional impairment, while higher age alone, did not predispose for delirium. Main precipitators were ARF and LD.

### Relationship between age and occurrence of delirium

Across most hospital populations, higher age is considered a main predisposing factor for delirium (9). In previous subgroup analyses from the same cohort, we found that age was a relevant predisposing in patients admitted to cardiology (25), neurology (12), and neurosurgery (13). By contrast, in our subgroups from stroke unit (11) and trauma care (26), similar to the presented GI- and HPB- cohort, age alone was not a relevant predisposing factor. This finding substantially supported the low-predisposition–severe-precipitator paradigm (6, 27), and identified ARF and LD as potential triggers for delirium in younger adults. In addition, this indicated that patients with dementia and functional impairment, as a part of geriatric syndrome (28), are at higher risk delirium, whereas patients who aged healthy have lower odds (29).

### Terminology: Delirium and encephalopathy

The term “delirium” subsumes various interchangeably used terms. For example, encephalopathy is a term frequently used by neurologists, while, the equivalent psychiatric term is delirium (30). This study used a pragmatic approach to avoid confusion with terminology, and deliberately did not subdivide the data. From a clinical perspective, the manifestations of what is currently termed delirious and encephalopathic syndrome are not as distinct that a subdivision would be expedient. That said, it should be stressed that pathomechanisms related to the underlying diseases vary among patients with delirious syndrome (31–33).

### Comparison to the literature in gastrointestinal- and hepato-pancreato-biliary-diseases

Several studies investigated delirium in specific HPB- conditions, such as post liver transplant delirium, precluding high internal validity (34). In these studies, it was shown that age, cerebrovascular disorders, cardiovascular, and underlying liver diseases contribute to delirium. Renal failure and infection were precipitating factors. Other studies have focused on surgical patients, for instance, in patients undergoing hepatectomy (35, 36). As in our cohort, delirium was associated with an overall increased hospital and ICU length of stay. Infections, ARF and transplantation mode were precipitating factors whereas age functions as predisposing factor. In patients managed in critical care and in patients with cirrhosis, ARF also has been described as a risk factor for delirium (37, 38). Our findings expand these findings to patients managed at a non-surgical and non-ICU GI- and HPB-diseases. Furthermore, it should be mentioned this was not a specifically hepatologic cohort. Therefore, the presented results provide an actual picture of the clinical reality in a tertiary hospital. Notably, it was possible to identify the cluster LD, ARF, and delirium to be associated with a high risk of death as well as the presence of liver cirrhosis in combination with precipitating factors and delirium. Despite this, functional impairment, not age, is the fundamental driver of delirium development in these patients. Furthermore, few studies to date have addressed the immediate and long-term consequences of delirium in this patient population (39–41). Based on our data, delirium is associated with loss of independence, as evidenced by frequent discharge to nursing homes even in younger patients.

### Mortality in patients with gastrointestinal- and hepato-pancreato-biliary-diseases and delirium

The odds of decease in the subgroup of GI-/HPB-patients with delirium was the highest of all non-surgical patients in the entire cohort of 39.432 patients (12, 42). Yet, the incidence of delirium was comparable to other studies (43, 44). The risk of decease in our analysis was found to be in mid-range, but markedly increased in patients with a GI-/HPB-disorder and delirium (18% versus 0.6%, OR 39.23) (43–45). The independent effect of delirium on mortality was previously examined and identified as a risk factor to decease even after covariate adjustment (17, 42, 46–48). In the present cohort, the odds to decease during hospitalization were more than fifteen-times higher in the presence of delirium and therefore seems much higher than previously assumed (44, 49). We expected a clear influence of LD on the occurrence of delirium and on the risk to decease (16). The occurrence of secondary delirium in LD is fatal for individuals affected and associated with a reduced 1-year-survival-rate (16). In our analysis, 40% of patients with delirium had LD (OR 2.22) and 49% suffered from cirrhosis (OR 2.11).

The setting of a tertiary hospital reveals more severe and advanced disease, which is a possible interpretation for the results in our population. However, a previous study showed that mortality rate in delirium in a more general population was not significant different between teaching hospitals and non-teaching hospitals (48). Recent studies have hypothesized that despite a reduction in delirium due to multi-component interventions, mortality and institutionalization were not altered, meaning that these endpoints may represent non-modifiable manifestations of delirium, and may be insensitive to a reduction in incident delirium (47, 50). This emphasizes that the delirium-associated risk of deceasing is interrelated to the underlying disease, disease severity and functional impairment.

### Clinical implications

Prevention of delirium is desirable for both patients and health care workers and can reduce health service costs (17). Hospital-service- specific risk profiles may help to identify patients at risk at an early stage. An important first step is to consider delirium in younger GI-/HPB-patients and vice versa to assess for HBP disease in young patients with unexplained delirium, to screen them adequately and to implement preventative strategies and multicomponent interventions (47, 51). Adequate training of all healthcare professionals in the recognition of delirium by means of bedside assessment methods, for example according to the approach used in the DELIR-PATH project (refer Supplementary material), would be a preliminary step. Furthermore, it is of high importance to avoid delirium-triggering/maintain drugs (52), and to provide sufficient pain treatment (53). High costs and nursing workload implied that delirium prevention strategies and coordinated treatment approaches will not only be of medical importance but will play a major role in healthcare-related cost reduction and resource allocation. In addition to healthcare-related costs, this could attenuate the detrimental long-term consequences of delirium (50, 51).

### Strengths and limitations

This study prospectively assessed predisposing and precipitating factors for delirium in acute GI-/HPB-patients with a large sample size, representative of a tertiary care center. Notably, all elderly patients were routinely assessed for delirium at admission, without any age limit, and, therefore, this study provided an actual picture of the clinical reality in a tertiary hospital. All data were electronically retrieved from patient charts. Relevant sociodemographic and medical parameters as well as comorbidities were recorded. However, baseline cognition and function, as well as severity of illness, e.g., Child-Pugh and Model of End Stage Liver Disease scores, and medication were not recorded. Due to the automated retrieval and sample size, compromises were required, e.g., the determination of delirium was based on DOS scores and a construct of delirium. Further, ICD-10 includes more than 13,000 codes and those had to be reduced with diagnostic clusters. Clusters may be heterogeneous based on the subheadings and not all codes could be included, rather a selection of delirium-relevant codes was chosen. Information may have been skewed or lost in this process. Nonetheless, these findings are novel and will require further confirmation in additional studies.

## Conclusion

Delirium is frequent in GI-/HPB-patients hospitalized in a tertiary hospital and associated with a significantly increased risk of death. Disease severity and functional impairment are the main drivers; importantly age *per se* appears not to contribute. Admissions from nursing homes or other hospitals are risk predictors and act as surrogate markers of disease severity and functional impairment. The presence of delirium has further serious consequences for the affected patients, as they more often require ICU, experience a longer hospital stay and are more frequently transferred to other hospitals or discharged to assisted living. Moreover, treating these patients incurs high health care costs and requires significant nursing resources. Establishing screening tools for identifying patients at-risk in advance, early diagnosis and development of treatment algorithms are therefore highly desirable.

## Data availability statement

The original contributions presented in this study are included in the article/Supplementary material, further inquiries can be directed to the corresponding author.

## Ethics statement

The study protocol was approved by the Ethics Committee of the Canton of Zurich and in compliance with Swiss federal law, a waiver of informed consent was granted by the Ethics Committee (KEK-ZH-Nr. PB-2016-01264).

## Author contributions

FFH, CMZ, and SB conceived the concept of this report. FFH and SB acquired the data. FFH, FRM, CMZ, RK, ARD, PS, JE, and SB conceived the critical appraisal of the method. FFH prepared the first draft of the manuscript. All authors provided edits and reviewed the manuscript for intellectual content.

## Abbreviations

ARF: acute renal failure
CNS: central nervous system
DCD: Degenerative cerebral disorders
DOS: Delirium observation screening scale
DSM-5: Diagnostic and Statistical Manual
ePA-AC: Electronic patient assessment - Acute care
GI: gastrointestinal
HPB: hepato-pancreato-biliary
IBD: Inflammatory bowel disease
IBS: Irritable bowel syndrome
ICD-10: International classification of diseases
ICU: Intensive care unit
LD: liver disease
LOS: Length of hospital stay
PB: pancreato-biliary.
