# Supplementary material for: Predisposing and precipitating risk factors for delirium in gastroenterology and hepatology: Subgroup analysis of 718 patients from a hospital-wide prospective cohort study
Source: Front Med (Lausanne). 2022 Nov 30;9:1004407. doi: 10.3389/fmed.2022.1004407 (PMC9747774; doi:10.3389/fmed.2022.1004407)
Supplement: Supplementary file 1 [file Data_Sheet_1.pdf]

Delirium in gastroenterology and hepatology: lessons from a prospective cohort study of 718 patients

Hildenbrand et al.

Supplementary Material

Methods

Determination of delirium

As part of the DELIR PATH project delirium was determined on the basis of the Delirium Observation Screening Scale (DOS) (Schuurmans et al., 2003) or the Diagnostic and Statistical Manual (DSM)-5 relevant parameters of the ePA-AC (D. S. Hunstein, B.; Rode, D.; Fiebig, M.; Dintelmann, Y., 2012). A patient was considered delirious if any of these tools indicated the presence of delirium at least once during the hospitalization. This construct accurately detects delirium in 97% of cases (Seiler et al., 2019).

Trained nursing staff educated in a 4-hour course with preceding prior eLearning performed screening. Training included case reports, state-of-the-art lectures in epidemiology, pathophysiology and in the diagnostic criteria of delirium. At the end of the course, the learning success was reviewed by a multiple choice test. The departments involved were continuously supported by the Delirium Task force, consisting of a consulting psychiatrist team dedicated to somatic in-hospital patient care, during the study`s initiation period and later on-demand.

Delirium Observation Screening Scale (DOS)

DOS is a 13-item scale validated to indicate delirium according to DSM-5 criteria. Items include disturbances of consciousness (1), attention (2-4), thought processes (5 and 6), orientation (7 and 8), memory (9), psychomotor behaviour (10, 11 and 13), and affect (12).

Symptoms are rated on a scale (0-1) as not existent (0), sometimes to always existent (1), and unable to assess (-). The cut-off score for delirium is ≥3 and values are aggregated throughout recordings. The DOS is administered three times daily during the first three days of admission for all elderly patient ≥65 years and in patients <65 years upon clinical suspicion of incident delirium. The cut-off of ≥ 3 is commonly used, as it has good diagnostic performance, with a sensitivity of 0.89, and a specificity of 0.88, and a high negative predictive value of almost 100% (Gemert van & Schuurmans, 2007; Yamanashi et al., 2021).

Electronic Patient Assessment-Acute Care (ePA-AC)

The ePA-AC is a 56 items nursing instrument administered daily assessing mobility, personal care and dressing, feeding, elimination, cognition and alertness, communication and interaction, sleeping, breathing, pain, pressure ulcers and wounds. The items reflecting the DSM 5 criteria of delirium were applied in an algorithm to determine the presence of delirium. The ePA-AC was applied in all patients without age restriction, and allowed us to rate the DSM-5 construct across all patients, even those not screened with DOS. The ePA-AC based instrument showed excellent agreement with the validated DOS cut-offs indicating that the overall specificity equal to those of the DOS (D. Hunstein, 2012).

Discharge disposition

Discharge destination was defined as the next setting to which a patient was discharged at the end of the hospitalization, which was obtained from the individual medical discharge report. These included patients' home (with or without long-term home care), an outside secondary care hospital (for physical aftercare or a psychiatric center), an acute rehabilitation center, nursing care home, or death (if patients died during the hospitalization).

Delirium management at the institution

The hospital followed an established management Standard Operating Procedure (SOP) for delirious patients. Primarily, non-pharmacological strategies are pursued, following well-established approaches for reality orientation for patients with cognitive disorders (Taulbee & Folsom, 1966). In addition, supportive means are applied by constant nurse caregivers, including adequate pain management, and provision of management of light and dark periods for restoration of the sleep-wake cycle. Throughout the present study, the management SOP remained unchanged.

Supplementary Table 1

Predisposing factors	ICD-10 Codes
Dementias / degenerative cerebral disorders	F00 Alzheimer’s disease<br>F01 vascular dementias<br>F02 Dementia due to elsewhere defined disorders<br>F03 Dementia not elsewhere defined<br>G30 Alzheimer’s disease<br>G31-.0 localized atrophies (frontal temporal dementia)<br>G31-.1-2 senile and alcohol-induced degenerations<br>G31.8-9 Degenerations<br>G32 degenerations due to elsewhere defined disorders
Substance use disorders	F10-19
Cirrhosis	K74 Fibrosis and cirrhosis of the liver<br>K70.3 Alcoholic cirrhosis of the liver<br>K71.7 Toxic liver disease with fibrosis and cirrhosis of the liver
**Precipitating factors**	
Liver disease	K70 Alcoholic liver disease<br>K71 Toxic liver disease<br>K72 Acute and subacute hepatic failure<br>K73 Hepatitis, not elsewhere classified<br>K74 Fibrosis and cirrhosis of the liver<br>K75 Other inflammatory liver diseases<br>K76 Other diseases of liver<br>K77 Liver disorders in diseases classified elsewhere
Acute renal failure	N17
**Functional Clusters**	
Decreased activity	
Decreased mobility	
Hearing impairment	
Visual impairment	

Supplementary Table 1: Diagnostic clusters with their respective included diagnoses according to the International Statistical Classification of Diseases and Related Health Problems 10th Revision (ICD-10).

Supplementary Table 2

ICD-10 Chapter	ICD-10 codes	N
Intestinal infectious diseases	A020, A045, A047, A081, A084, A090, A181	11
Other bacterial diseases	A402, A410, A414, A4151, A4158, A419, A46, A493, A498	12
Viral infections characterized by skin and mucous membrane lesions	B023, B028	2
Viral hepatitis	B159, B179, B181	4
Other viral diseases	B338	2
Mycosis	B3781	1
Protozoal diseases	B59	1
Helminthiasis	B675	2
Other infectious diseases	B99	3
Malignant neoplasms of digestive organs	C151, C153, C154, C155, C158, C160, C162, C163, C187, C20, C220, C221, C240, C241, C250, C258, C504, C786, C787, C822	89
In situ neoplasms	D001, D010, D011, D012	5
Benign neoplasms	D120, D122, D126, D128, D132, D135	20
Neoplasms of uncertain or unknown behaviour	D371, D3770, D45	3
Nutritional anaemias	D508	6
Aplastic and other anaemias	D6118	1
Coagulation defects, purpura and other haemorrhagic conditions	D6830	1
Other diseases of blood and blood-forming organs	D728	1
Malnutrition	E440	1
Obesity and other hyper alimentation	E6601, E6602	5
Metabolic disorders	E748	1
Disorders of mineral metabolism	E8358	1
Other disorders of fluid, electrolyte and acid-base balance	E871	1
Neurotic, stress-related and somatoform disorders	F4001, F432	2
Extrapyramidal and movement disorders	G2011	1
Demyelinating diseases of the central nervous system	G372	1
Episodic and paroxysmal disorders	G403, G448	2
Ischaemic heart diseases	I214, I2511	3
Other forms of heart disease	I471	1
Diseases of arteries, arterioles and capillaries	I780	1
Diseases of veins, lymphatic vessels and lymph nodes, not elsewhere classified	I820, I841, I844, I848, I864, I868, I891	8
Influenza and pneumonia	J188, J189	2
Other diseases of upper respiratory tract	J36	1
Chronic lower respiratory diseases	J4403, J4481	2

Diseases of oesophagus, stomach and duodenum	K20, K210, K221, K222, K223, K225, K226, K227, K250, K253, K254, K257, K260, K264, K283, K284, K290, K296, K311, K315, K3182, K3188	98
Hernia	K420, K430, K449	3
Noninfective enteritis and colitis	K500, K501, K5082, K510, K513, K515, K518, K519, K528, K529	36
Other diseases of intestines	K550, K5522, K5582, K566, K567, K5711, K5730, K5731, K5732, K5733, K589, K590, K598, K620, K621, K631, K633, K635	41
Diseases of peritoneum	K650, K658	5
Diseases of liver	K701, K703, K704, K710, K711, K718, K719, K720, K721, K740, K743, K744, K746, K750, K754, K758, K766, K768	158
Disorders of gallbladder, biliary tract and pancreas	K8000, K8001, K8020, K8021, K8031, K8050, K8051, K8080, K8081, K830, K831, K838, K8500, K8501, K8510, K8511, K8520, K8530, K8580, K8590, K861, K863, K868	112
Other diseases of the digestive system	K904, K920, K921, K922	31
Dorsalgia	M5487	1
Urinary tract infection, site not specified	N390	1
Malfunction of external stoma of urinary tract	N995	1
Other maternal diseases classifiable elsewhere but complicating pregnancy, childbirth and the puerperium	O996	1
Other congenital malformations of the digestive system	Q393, Q447, Q453	4
Symptoms and signs involving the digestive system and abdomen	R100, R101, R104, R11, R130, R18	13
Symptoms and signs involving cognition, perception, emotional state and behaviour	R418	1
General symptoms and signs	R5080, R571	2
Injuries to the head	S0123, S065	2
Injuries to the abdomen, lower back, lumbar spine and pelvis	S3201, S3650, S3654	3
Effects of foreign body entering through natural orifice	T181, T182	2
Burns and corrosions	T286	1
Poisoning by drugs, medicaments and biological substances	T391	1
Complications of surgical and medical care, not elsewhere classified	T846, T855, T8588	4

Supplementary Table 2: Subjects by main diagnoses. Overview of available ICD-10 chapters and ICD-10 codes. N= number of patients in each ICD-10 chapter

Supplementary Figure legends

Supplementary Figure 1: Distribution by main diagnosis of 718 patients in the study population with and without delirium. Data were calculated on the proportion of patients in each ICD-10 diagnosis group. **Abbrevations**: IBD: Inflammatory bowel disease IBS: Irritable bowel syndrom GI: gastrointestinal

Supplementary Figure 2: Distribution of delirium by main diagnosis. Data were calculated on the proportion of patients in the delirium group. **Abbrevations:** IBS : Irritable bowel syndrome, IBD: Inflammatory bowel disease, DCD: Degenerative cerebral disorders, ARF: Acute renal failure

References

Gemert van, L. A., & Schuurmans, M. J. (2007). The Neecham Confusion Scale and the Delirium Observation Screening Scale: capacity to discriminate and ease of use in clinical practice. *BMC Nurs*, *6*, 3. https://doi.org/10.1186/1472-6955-6-3

Hunstein, D. (2012). ePAAC©: ergebnisorientiertes PflegeAssessment AcuteCare (Version 2.0). 1-72.

Hunstein, D. S., B.; Rode, D.; Fiebig, M.; Dintelmann, Y. (2012). ePAAC: ergebnisorientiertes PflegeAssessment AcuteCare (Version 2.0).

Schuurmans, M. J., Shortridge-Baggett, L. M., & Duursma, S. A. (2003). The Delirium Observation Screening Scale: a screening instrument for delirium. *Res Theory Nurs Pract*, *17*(1), 31-50.

Seiler, A., Schubert, M., Hertler, C., Schettle, M., Blum, D., Guckenberger, M., Weller, M., Ernst, J., von Kanel, R., & Boettger, S. (2019). Predisposing and precipitating risk factors for delirium in palliative care patients. *Palliat Support Care*, 1-10. https://doi.org/10.1017/S1478951519000919

Taulbee, L. R., & Folsom, J. C. (1966). Reality orientation for geriatric patients. *Hosp Community Psychiatry*, *17*(5), 133-135.

Yamanashi, T., Iwata, M., Crutchley, K. J., Sullivan, E. J., Malicoat, J. R., Anderson, Z. M., Marra, P. S., Chang, G., Kaneko, K., Shinozaki, E., Lee, S., & Shinozaki, G. (2021). New Cutoff Scores for Delirium Screening Tools to Predict Patient Mortality. *J Am Geriatr Soc*, *69*(1), 140-147. https://doi.org/10.1111/jgs.16815
