# Supplementary figures and images for: Predisposing and precipitating risk factors for delirium in gastroenterology and hepatology: Subgroup analysis of 718 patients from a hospital-wide prospective cohort study
Source: Front Med (Lausanne). 2022 Nov 30;9:1004407. doi: 10.3389/fmed.2022.1004407 (PMC9747774; doi:10.3389/fmed.2022.1004407)

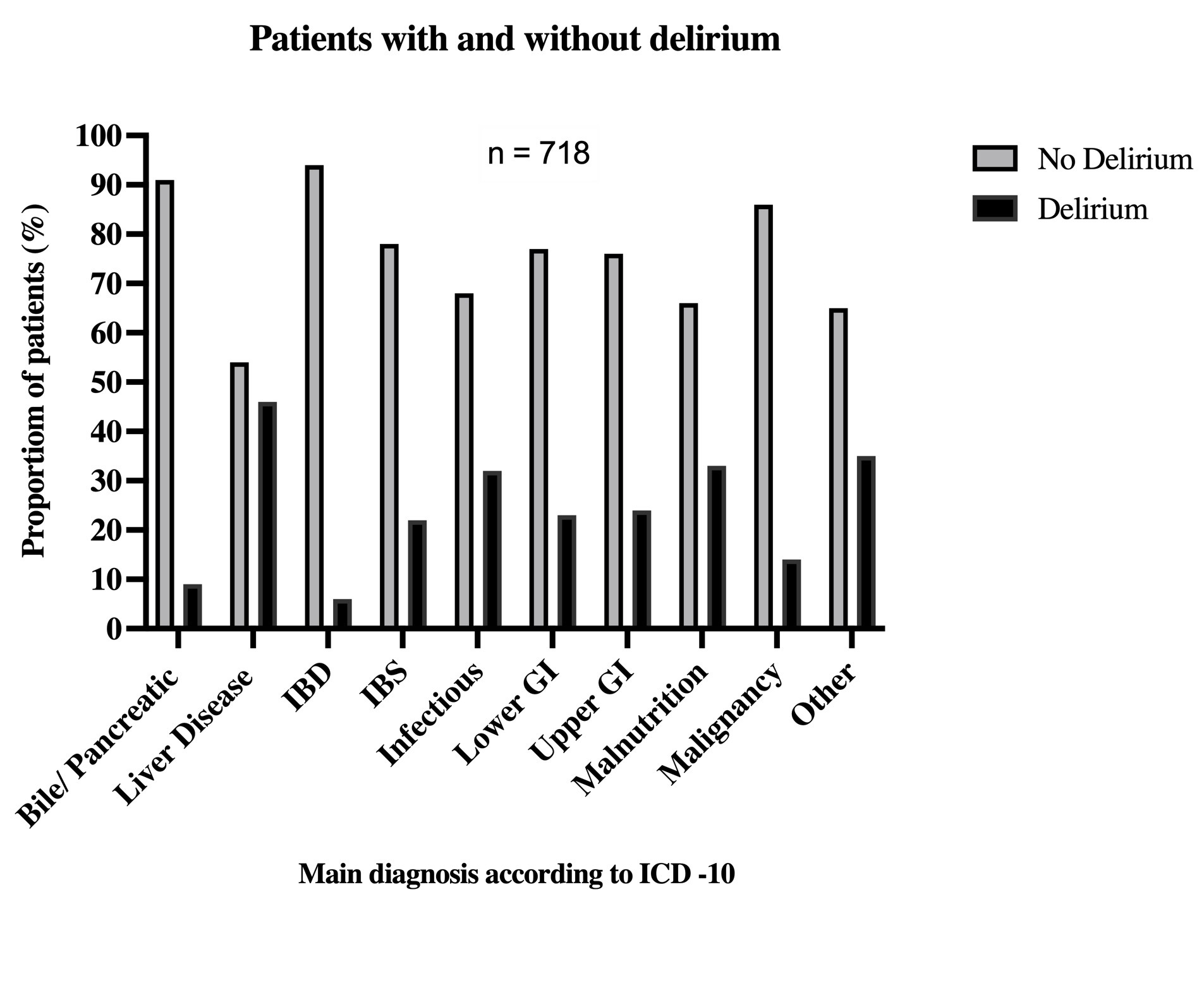

Supplement: Supplementary file 2 [file Image_1.jpeg]

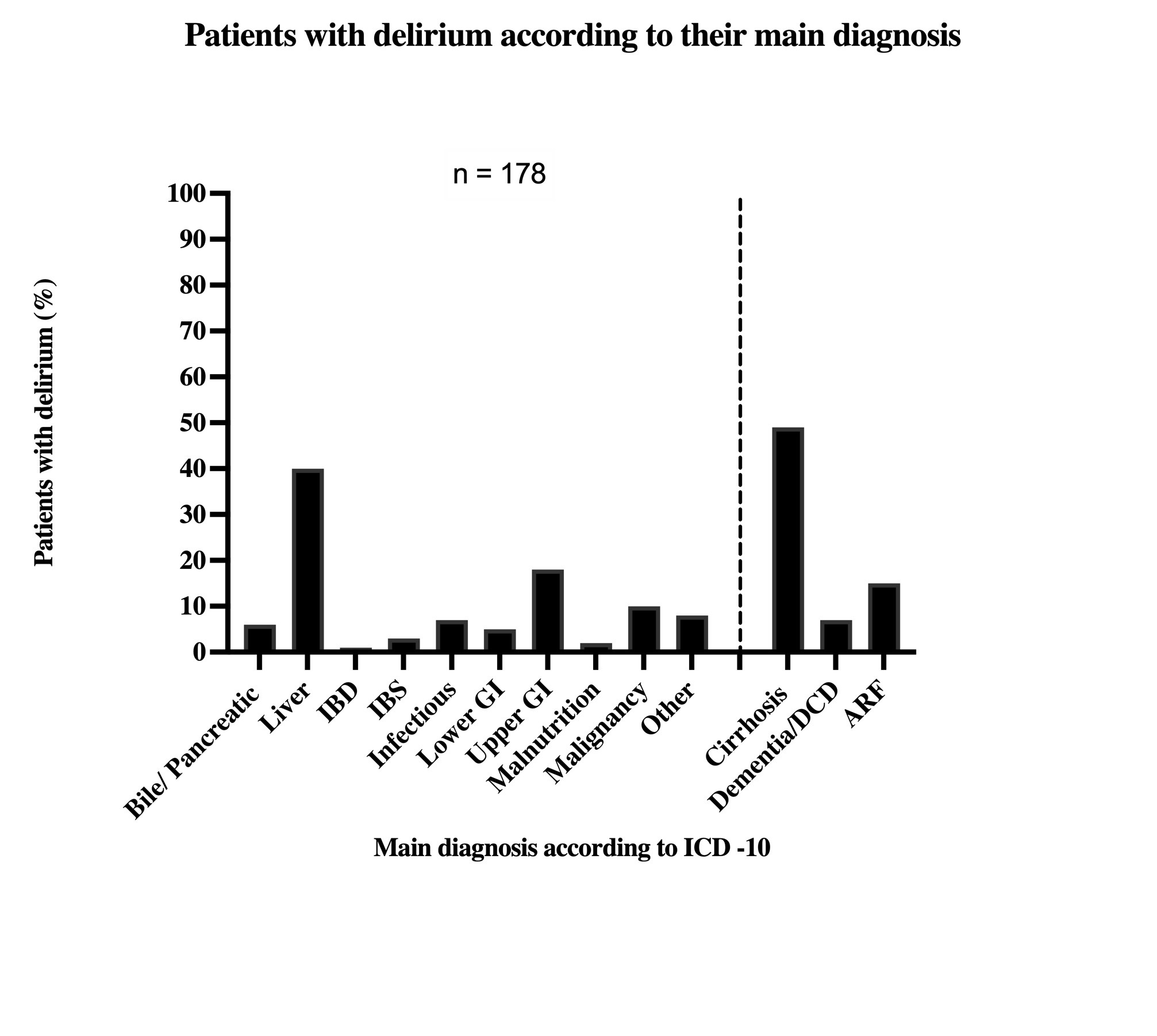

Supplement: Supplementary file 3 [file Image_2.jpeg]
